# Increased diaphragm echodensity correlates with postoperative pulmonary complications in patients after major abdominal surgery: a prospective observational study

**DOI:** 10.1186/s12890-022-02194-6

**Published:** 2022-11-04

**Authors:** Xin Fu, Zhen Wang, Luping Wang, Guangxuan Lv, Yisong Cheng, Bo Wang, Zhongwei Zhang, Xiaodong Jin, Yan Kang, Yongfang Zhou, Qin Wu

**Affiliations:** Department of Critical Care Medicine, West China Hospital, Sichuan University, Chengdu, China

**Keywords:** Ultrasonography, Diaphragm echodensity, Major abdominal surgery, Postoperative pulmonary complications, Mechanical ventilation, Intensive care

## Abstract

**Background:**

Associated with increased morbidity and mortality, postoperative pulmonary complications (PPCs) often occur after major abdominal surgery. Diaphragmatic dysfunction is suggested to play an important role in the development of PPCs and diaphragm echodensity can be used as an indicator of diaphragm function. This study aimed to determine whether diaphragm echodensity could predict the occurrence of PPCs in patients after major abdominal surgery.

**Methods:**

Diaphragm ultrasound images of patients after major abdominal surgery were collected during spontaneous breathing trials. Echodensity was quantified based on the right-skewed distribution of grayscale values (50th percentile, ED50; 85th percentile, ED85; mean, EDmean). Intra- and inter-analyzer measurement reproducibility was determined. Outcomes including occurrence of PPCs, reintubation rate, duration of ventilation, and length of ICU stay were recorded.

**Results:**

Diaphragm echodensity was measured serially in 117 patients. Patients who developed PPCs exhibited a higher ED50 (35.00 vs. 26.00, *p* < 0.001), higher ED85 (64.00 vs. 55.00, *p* < 0.001) and higher EDmean (39.32 vs. 33.98, *p* < 0.001). In ROC curve analysis, the area under the curve of ED50 for predicting PPCs was 0.611. The optimal ED50 cutoff value for predicting the occurrence of PPCs was 36. According to this optimal ED50 cutoff value, patients were further divided into a high-risk group (ED50 > 36, n = 35) and low-risk group (ED50 ≤ 36, n = 82). Compared with the low-risk group, the high-risk group had a higher incidence of PPCs (unadjusted *p* = 0.003; multivariate-adjusted *p* < 0.001).

**Conclusion:**

Diaphragm echodensity can be feasibly and reproducibly measured in mechanically ventilated patients. The increase in diaphragm echodensity during spontaneous breathing trials was related to an increased risk of PPCs in patients after major abdominal surgery.

**Supplementary Information:**

The online version contains supplementary material available at 10.1186/s12890-022-02194-6.

## Background

Postoperative pulmonary complications (PPCs), ranging from 6 to 80%, are the leading cause of increased morbidity and mortality after chest and abdominal surgery in adults, particularly within the first postoperative week [1, 2]. To promptly identify high-risk patients, several scoring systems have been developed to predict the development of PPCs, including the Assess Respiratory Risk in Surgical Patients in Catalonia score (ARISCAT score) [1], the Local Assessment of Ventilatory Management During General Anesthesia for Surgery (LAS VEGAS) risk score [3], and the Score for Prediction of Postoperative Respiratory Complications (SPORC) [4]. However, because of diverse perioperative factors and variability across a wide range of surgical settings, the scoring systems used to predict PPCs are of limited clinical use.

It has been suggested that diaphragmatic dysfunction, including injury of the diaphragm bundle secondary to surgery and mechanical ventilation, is one of the main causes of PPCs after major abdominal surgery. This raises the possibility of predicting PPCs via diaphragmatic dysfunction evaluation. It has been reported that diaphragm thickness and diaphragm thickness fraction can be used to predict PPCs in patients undergoing Robot-Assisted Laparoscopic Prostatectomy [5]. However, the diaphragm thickness fraction does not reflect diaphragm bundle damage, which is believed as a real early injury for diaphragmatic dysfunction [6]. The use of quantitative ultrasound has been proposed in pulmonary ultrasound and skeletal muscle ultrasound [7, 8], and diaphragm echodensity, as a newer quantitative technique, can quantify the damage of the diaphragm bundle and reflect the degree of damage to the diaphragm bundle [6]. Previous studies have reported that increases in diaphragm echodensity during the early course of mechanical ventilation were associated with prolonged mechanical ventilation [9]. We hypothesize that increased diaphragm echodensity is related to a higher risk of PPCs in patients after major abdominal surgery and that diaphragm echodensity can predict the occurrence of PPCs.

## Measurements and main results

### Study design and participants

A prospective cohort study was performed in the surgical intensive care unit of West China Hospital. The study procedures and protocol were approved by the West China Hospital Research Ethics Committee (No. 2021S857).

Patients 18 years of age or older who underwent major abdominal surgery were eligible for inclusion. Patients were allocated into two groups based on whether PPCs occurred or not. Participants were excluded if they were pregnant or lactating, or had genetic and/or chronic skeletal muscle disorders (e.g. motor nerve disorders), or had unilateral diaphragmatic pathology (e.g. phrenic nerve or spinal cord injury), or had participated in other studies.

PPCs were defined as atelectasis, pneumonia, bronchospasm, pleural effusion, pulmonary edema, and respiratory failure [10]. A standard definition for PPCs was determined by the ESA-ESICM joint task force on perioperative outcome measures [11]. All complications are defined in detail in Additional Table 1.

**Table 1:** Characteristics of enrolled patients

Characteristics	All (n = 117)	Study group		P value
		**PPCs (n = 56)**	**Non-PPCs (n = 61)**	
Age, mean (SD), y	57.57 (15.46)	60.64 (16.53)	54.41 (13.93)	0.030
Male, n (%)	78.00 (66.67)	38.00 (67.86)	40.00 (65.57)	0.794
BMI, mean (SD)	22.47 (4.24)	22.43 (3.67)	22.52 (4.77)	0.911
ARISCAT score, mean (SD)	38.39(16.74)	47.93 (16.04)	30.07 (12.11)	< 0.001
ASA class, n (%)				< 0.001
Grade 2	55.00 (47.01)	14.00 (25)	41.00 (67.21)	
Grade 3	56.00 (47.86)	40.00 (71.43)	16.00 (26.23)	
Grade 4	6.00 (5.13)	2.00 (3.57)	4.00 (6.56)	
APACHE II score, mean (SD)	13.29 (7.39)	14.75 (7.87)	11.95 (6.77)	0.041
Comorbidity, n (%)				
Cardiovascular disease	20.00 (17.09)	15.00 (26.79)	5.00 (8.20)	0.008
Diabetes mellitus	9.00 (7.69)	4.00 (7.14)	5.00 (8.20)	1.000
COPD	4.00 (3.42)	3.00 (5.36)	1.00(1.64)	0.551
Hyperlipoidemia	5.00 (4.27)	5.00 (8.93)	0.00 (0.00)	0.054
Hypertension	17.00 (14.53)	11.00 (19.64)	6.00 (9.84)	0.133
Active cancer	49.00 (41.88)	21.00 (37.50)	28.00 (45.90)	0.357
Chronic renal disease	38.00 (32.48)	22.00 (39.29)	16.00 (26.23)	0.132
Chronic liver disease	48.00 (41.03)	22.00 (39.29)	26.00 (42.62)	0.714
Chronic bronchitis	4.00 (3.42)	4.00 (7.14)	0.00 (0.00)	0.106
Type of surgery, n (%)				
Emergency surgery	42.00 (35.90)	31.00 (55.36)	11.00 (18.03)	< 0.001
Planned surgical procedure, n (%)				0.318
Resection of colon, rectum, or small bowel	19.00 (16.24)	12.00 (21.43)	7.00 (11.48)	
Resection of liver, pancreas, or gall bladder	69.00 (58.98)	32.00 (57.15)	37.00 (60.66)	
Resection of stomach	5.00 (4.27)	3.00 (5.36)	2.00 (3.28)	
Other intraperitoneal surgery	24 (20.51)	9(16.07)	15(24.59)	
**Incision type, n (%)**				0.803
Midline laparotomy	61.00 (52.14)	31.00 (55.36)	30.00 (49.18)	
Bilateral or unilateral subcostal	23.00 (19.66)	12.00 (21.43)	11.00 (18.03)	
Transverse abdominal	1.00 (0.85)	0.00 (0.00)	1.00 (1.64)	
Laparoscopic or lower abdominal	13.00 (11.10)	5.00 (9.00)	8.00 (13.10)	
Other	19.00 (16.24)	8.00 (14.29)	11.00 (18.03)	
Length of procedure, mean (SD), mins	199.84 (189.01)	184.89 (184.10)	213.18 (196.95)	0.425
Diaphragm echodensity, median (IQR)				
ED50	30.00 (22.00–38.00)	35.00 (22.00–44.00)	26.00 (21.00–35.00)	< 0.001
EDmean	35.33 (26.82–43.63)	39.32 (29.88–48.37)	33.98 (26.14–41.37)	< 0.001
ED85	58.00 (46.00–72.00)	64.00 (49.00–76.00)	55.00 (44.00–63.00)	< 0.001
Diaphragm mobility, mean (SD), cm	1.10 (0.44)	1.12 (0.41)	1.08 (0. 47)	0.696
Diaphragm thickness, mean (SD), cm	0.23 (0.07)	0.23 (0.07)	0.22 (0.06)	0.354
Length of ICU stay, mean (SD), day	6.48 (6.21)	8.11 (7.39)	4.98 (4.51)	0.008
Length of hospital stay, mean (SD), day	17.37 (15.49)	20.21 (17.81)	14.75 (12.76)	0.061
VFD-28, mean (SD), day	25.35 (3.78)	24.55 (4.17)	26.05 (3.32)	0.035
Mortality, n (%)	6.00 (5.13)	5.00 (8.93)	1.00 (1.64)	0.103
Reintubation, n (%)	7.00 (5.98)	6.00 (10.71)	1.00 (1.64)	0.093
**Ventilatory support after extubation, n (%)**				0.001
COT	68.00 (58.12)	26.00 (46.43)	42.00 (68.85)	
HFNC	14.00 (11.86)	4.00 (7.14)	10.00 (16.13)	
NIPPV	35.00 (29.66)	26.00 (46.43)	9.00 (14.52)	

Abbreviations: PPCs, Postoperative Pulmonary Complications; non-PPCs, no Postoperative Pulmonary Complications; BMI, Body Mass Index; ARISCAT score, Assess Respiratory Risk in Surgical Patients in Catalonia score; APACHE II, Acute Physiology and Chronic Health Evaluation; ASA class, American Society of Anesthesiologists class; COPD, chronic obstructive pulmonary disease; IQR, interquartile range; VFD-28, Ventilation Free Day of 28 days; ICU, intensive care unit; COT, conventional oxygen therapy; HFNC, high flow nasal cannula; NIPPV, noninvasive positive pressure ventilation

### Diaphragm echodensity measurement

Diaphragm ultrasound images were collected during the spontaneous breathing trial (SBT). During this period, the patient received the same ventilator parameters, which could minimize the impact of the ventilator on the measurement of the diaphragm index, and the index monitoring was more accurate. The method used to collect diaphragm ultrasound images to measure diaphragm thickness and mobility was described in the original study [12, 13]. To standardize ultrasound gain and frequency for echodensity measurements, B-mode images were obtained after restoring the ultrasound device setting to start-up, preset default values (frame rate 34 Hz, depth 5 cm, DB gain 50, Focus:2.8 cm, time-gain compensation: 50%). The same ultrasound machines (Philips Medical Capital, Netherlands) were used during the whole study period. Diaphragm echodensity was quantified by performing a grayscale histogram analysis in ImageJ (National Institutes of Health, Bethesda, MD, USA). For grayscale histogram analysis to quantify echodensity, the value range was determined using the trace method by selecting the largest free form area devoid of artifacts between (but excluding) the pleural and peritoneal membranes [14]. A grayscale frequency histogram was generated for the selected region. The distribution of echodensity for the selected region was right-skewed and was quantified using two different parameters: the 50th percentile (ED50) and the 85th percentile (ED85). The 50th and 85th percentile thresholds were deemed to represent the center and upper tail of the grayscale distribution, respectively. Meanwhile, the mean values of diaphragm echodensity (EDmean) were also included in the study. The images were analyzed using a single frame. In order to avoid the bias caused by subjective bias, we selected two observers who were blinded to the patient grouping to measure the diaphragm echodensity.

### Data collection

The age, body mass index, heart rate, blood pressure, comorbidities, admission diagnosis, Acute Physiology and Chronic Health Evaluation II (APACHE II) score, ventilator setting, arterial blood gas analysis, ASA score, and ARISCAT score were collected from the medical records system for each patient. The following outcomes were also collected: the number of ventilator-free days at day 28, reintubation rate, ICU mortality, hospital mortality, ED50, ED85, EDmean, diaphragm thickness, and diaphragm mobility. Liberation from ventilation was defined as separation from the ventilator (extubation or tracheostomy mask breathing) for 24 h without resumption of invasive ventilatory support during ICU admission [2].

### Statistical analysis

We performed a power analysis on the primary outcome, and when the sample size was 117, the power of the outcome measure was greater than 90%. This was based on a two-sided significance level of 5%. Continuous variables were expressed as the mean values (± standard deviation) or median values with interquartile ranges (IQR), according to the distribution (Shapiro–Wilk test). Discrete variables were expressed as percentage values. Categorical variables were compared between the PPCs and non-PPCs groups using the chi-square test or Fisher’s exact test. For continuous variables, we used an independent samples t test or Wilcoxon rank sum test. To assess the repeatability and reproducibility of echodensity measurements, the principal analyzer trained a secondary analyzer using 70 randomly selected images. Intra-analyzer repeatability and inter-analyzer reproducibility of ED50 (ED85 and EDmean) were quantified by the method of Bland and Altman [24] and paired-samples t test. At the same time, to assess whether echodensity measurements were affected by the timing of the respiratory cycle, the images closest to end-inspiration and end-expiration were captured, and echodensity was measured in 35 random videos covering the entire respiratory system.

To determine the ability of diaphragm echodensity to predict the occurrence of PPCs after abdominal surgery, receiver operator characteristic (ROC) curve analysis was performed. The value with the highest specificity and sensitivity was designated the optimal cutoff value. After dividing the patients into two groups according to the optimal diaphragm echodensity cutoff value for predicting PPCs (i.e., low-risk group vs. high-risk group), we compared the occurrence of PPCs between high-risk and low-risk patients. The ability of low diaphragm echodensity to predict the occurrence of PPCs was evaluated by a multivariate-adjusted odds ratio. The results were reported as odds ratios (ORs) with 95% CIs.

The relationship between the clinical characteristics (including diaphragm echodensity) and PPCs was assessed by logistic regression. Only variables both statistically associated with the primary outcome in bivariable tests (at *p* < 0.05) and deemed clinically relevant were included in multivariable models that assessed variables associated with the occurrence of PPCs. No interaction terms were included in modeling. At each step of multivariable modeling, forward elimination of variables not statistically significant was used to reach a final parsimonious model. A *p*-value of < 0.05 was considered to denote statistical significance.

Statistical analyses were conducted using IBM SPSS version 26.0.0 (IBM Corp., Armonk, NY, USA), MedCalc Software version 20.0.3 (MedCalc Software bvba, Mariakerke, Belgium), and GraphPad Prism version 8 (GraphPad Software Inc., San Diego, CA, USA).

## Results

### Participants

**Fig. 1:**
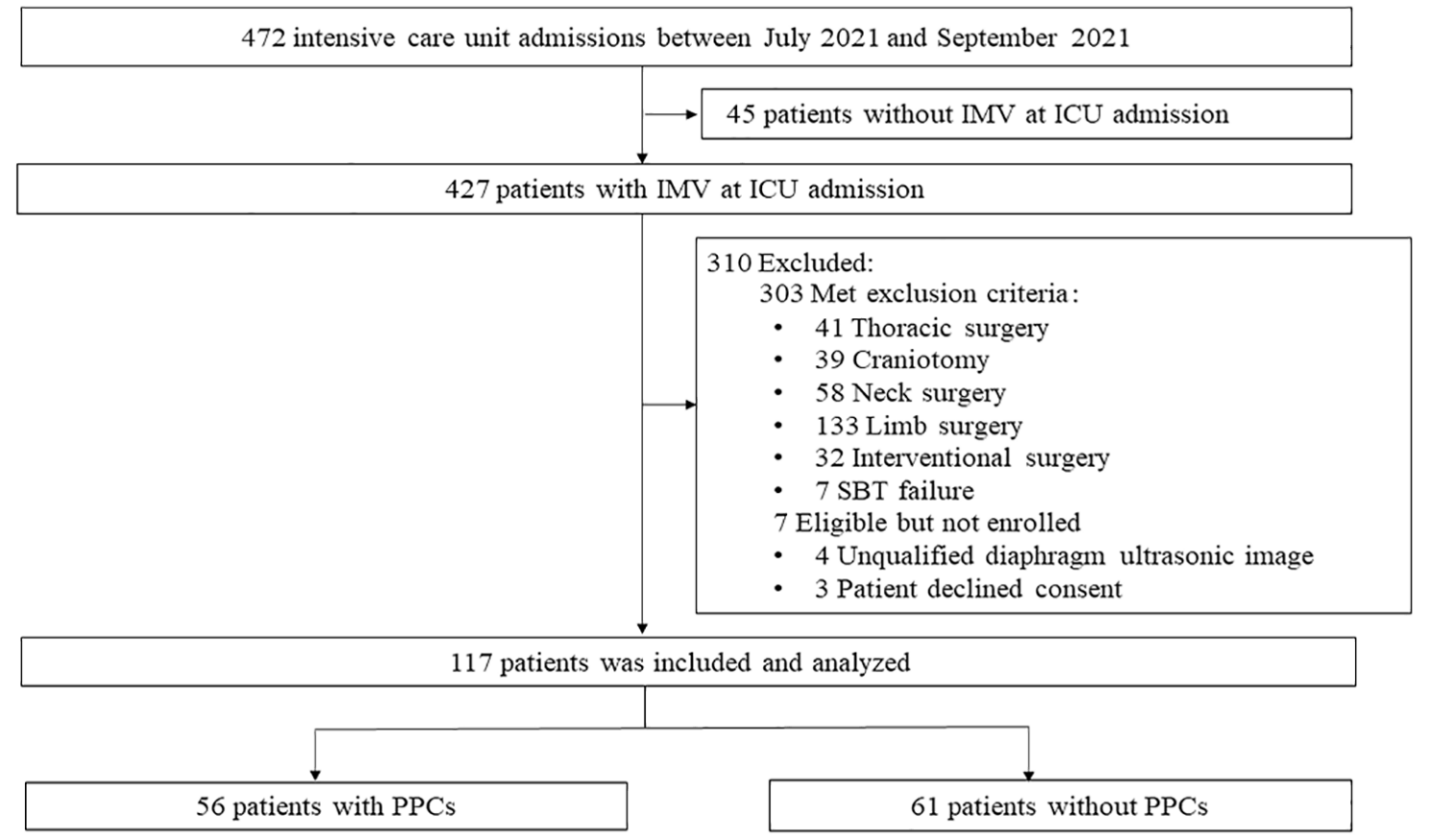
Study flowchart. PPCs: postoperative pulmonary complications; COT: conventional oxygen therapy; IMV: Invasive Mechanical Ventilation; SBT: spontaneous breathing trial

In total, 472 patients were screened for enrollment between July 14, 2021, and September 1, 2021. Of them, 310 (87.32%) patients were excluded because they had nonabdominal surgery before ICU admission, and 45 (14.52%) were excluded because they were extubated before ICU admission. The remaining 117 patients were enrolled for final analysis (Fig. 1).

According to the occurrence of PPCs, the whole cohort was divided into the PPCs group (56, 47.86%) and the non-PPCs group (61, 52.14%). At enrollment in the study, baseline comparison of the PPCs and non-PPCs participants revealed that the PPCs group was older (60.64 ± 16.53 in the PPCs group vs. 54.41 ± 13.93 in the non-PPCs group, *p* = 0.030), had a higher ARISCAT score (47.93 ± 16.04 in the PPCs group vs. 30.07 ± 12.11 in the non-PPCs group, *p* < 0.001) and had a higher APACHE II score (14.75 ± 7.87 in the PPCs group vs. 11.95 ± 6.77 in the non-PPCs group, *p* = 0.041) (Table 1). According to the basic condition of patients before surgery, ASA classes were distributed from 2 to 4, and the ASA class of the PPCs group was higher than that of the non-PPCs group (*p* < 0.001). A low proportion of participants were admitted following emergency surgery (31 [55.36%] in the PPCs group, 11 [18.03%] in the non-PPCs group, *p* < 0.001], with resection of the liver, pancreas, or gallbladder being most common, and midline laparotomy being the most common surgical incision type group. In the whole cohort, active cancer comorbidities (including gastric carcinoma, gallbladder adenocarcinoma, and rectum adenocarcinoma) were the most common, followed by chronic liver disease. In addition, 68 (58.12%) patients received conventional oxygen therapy (COT), 14 (11.97%) patients received high flow nasal cannula (HFNC), and 35 (29.91%) patients received noninvasive positive pressure ventilation (NIPPV). There were significant differences in the method of ventilatory support after extubation between the PPCs group and the non-PPCs group (*p* = 0.001) (Table 1).

The intraoperative and postoperative management data between the PPCs group and the non-PPCs group were compared in Table 2, Additional Table 2 and Additional Table 3. Patients who developed PPCs exhibited higher pH and lower PaO_2,_ upon ICU admission (pH: 7.38 ± 0.05 in the PPCs group vs. 7.36 ± 0.05 in the non-PPCs group, *p* = 0.024) (PaO_2_: 115.71 ± 39.97 in the PPCs group vs. 146.18 ± 36.18 in the non-PPCs group, *p* < 0.001). The PaO_2,_ which was measured at the end of SBT in the PPCs group, was also lower (112.82 ± 46.07 in the PPCs group vs. 145.86 ± 31.45 in the non-PPCs group, *p* < 0.001). Moreover, patients in the PPCs group showed a higher WBC count (12.39 ± 5.85 in the PPCs group vs. 9.27 ± 4.42 in the non-PPCs group, *p* = 0.002) and a higher CRP level (92.24 ± 107.8 in the PPCs group vs. 32.27 ± 70.45 in the non-PPCs group, *p* = 0.002).

**Table 2:** Intraoperative and postoperative management

Characteristics	All (n = 117)	Study group		P value
		**PPCs (n = 56)**	**Non-PPCs (n = 61)**	
Intraoperative management				
Type of intraoperative fluid, mean (SD), ml				
Crystalloid	1905.25 (1314.06)	1824.11 (1272.29)	1970.00 (1376.88)	0.554
Colloid*	945.14 (607.14)	869.70 (482.01)	1008.97 (702.61)	0.339
Blood transfusion				
CRCs, mean (SD), u	6.00 (4.15)	4.87 (3.23)	7.44 (4.90)	0.064
Plasma, mean (SD), ml	823.44 (806.03)	563.89 (308.6)	1157.14 (1123.16)	0.040
Intraoperative blood loss, mean (SD), ml	414.15 (815.46)	348.57 (534.5)	479.51 (1017.79)	0.392
Use of vasoactive drugs**				
Metaraminol, n (%)	64.00 (54.7)	32.00 (57.14)	32.00 (52.46)	0.374
Total Dose, mg	0.63 (0.63)	0.71 (0.65)	0.55 (0.63)	0.323
Ephedrine, n (%)	39.00 (33.33)	16.00 (28.57)	23.00 (37.70)	0.198
Total Dose, mg	8.06 (6.68)	8.31 (8.46)	7.89 (5.51)	0.851
Norepinephrine, n (%)	36.00 (30.77)	22.00 (39.29)	14.00 (22.95)	0.043
Total Dose, mg	1.76 (2.11)	1.73 (1.88)	1.80 (2.56)	0.927
Epinephrine, n (%)	4.00 (3.42)	1.00 (1.79)	3.00 (4.92)	0.342
Total Dose, mg	101.05 (95.36)	3.00 (0.00)	133.73 (108.53)	0.406
Use of opioids, mean (SD), µg**				
Remifentanil	1463.84 (809.02)	1534.51 (846.83)	1400.01 (781.54)	0.651
Sufentanil	43.91 (22.76)	44.85 (21.93)	43.60 (24.48)	0.149
Use of muscle relaxant, mean (SD), mg**				
Vecuronium bromide	6.06 (4.95)	3.65 (4.74)	7.67 (6.35)	0.507
Rocuronium Bromide	82.96 (33.84)	77.25 (30.76)	99.29 (41.68)	0.851
Atracurium	27.71 (20.44)	26.75 (20.67)	28.57 (20.56)	0.577
Succinylcholine	76.79 (18.28)	76.88 (21.87)	76.67 (16.33)	0.985
Intraoperative monitoring***				
pH	7.40 (0.10)	7.38 (0.13)	7.41 (0.04)	0.113
PaO_2_, mmHg	220.76 (90.69)	208.75 (91.30)	232.31 (90.34)	0.329
PaCO_2_, mmHg	37.42 (6.13)	37.40 (6.78)	37.45 (5.56)	0.962
Lactate, mmol/L	1.56 (1.96)	1.62 (2.70)	1.29 (0.52)	0.388
Postoperative monitoring				
Fluid delivery postoperative day 1, mean (SD), ml	2031.97 (1098.29)	2000.27 (1105.54)	2061.07 (1109.06)	0.767
ABG at ICU admission				
pH	7.35 (0.04)	7.38 (0.05)	7.36 (0.05)	0.024
PaO_2_, mmHg	125.37 (29.74)	115.71 (39.97)	146.18 (36.18)	< 0.001
PaCO_2_, mmHg	41.12 (4.95)	37.07 (6.31)	39.19 (6.07)	0.066
Lactate, mmol/L	1.68 (0.42)	2.01 (1.55)	2.77 (2.28)	0.035
ABG when the SBT ends				
pH	7.38 (0.05)	7.40 (0.05)	7.38 (0.05)	0.063
PaO_2_, mmHg	112.60 (13.20)	112.82 (46.07)	145.86 (31.45)	< 0.001
PaCO_2_, mmHg	41.95 (2.95)	38.82 (5.28)	38.81 (5.19)	0.986
Blood routine examination at ICU admission				
WBC, 10^9/L	10.96 (4.66)	12.39 (5.85)	9.27 (4.42)	0.002
Hemoglobin, mg/L	111.50 (4.57)	107.59 (31.79)	116.70 (19.81)	0.069
Biochemical examination at ICU admission				
Serum albumin, g/L	33.28 (2.64)	32.39 (7.19)	35.21 (7.22)	0.037
Serum creatinine, µmol/L	217.17 (325.87)	105.41 (141.33)	70.92 (39.27)	0.082
CRP, mg/L	61.01 (99.48)	92.24 (107.8)	37.27 (70.45)	0.002
PCT, ng/ml	1.39 (3.82)	2.16 (7.00)	0.53 (1.13)	0.090
Antibiotic delivery, n (%)***				
Imipenem	19.00 (16.24)	15.00 (26.79)	4.00 (6.56)	0.003
Penicillins and 1st-2nd generation cephalosporins	74.00 (63.25)	28.00 (50)	46.00 (75.41)	0.004
3rd generation cephalosporins/macrolides	15.00 (12.82)	11.00 (19.64)	4.00 (6.56)	0.034
Tetracyclines	7.00 (5.98)	6.00 (10.71)	1.00 (1.64)	0.172
Polypeptide antibiotic	8.00 (6.84)	7.00 (12.5)	1.00 (1.64)	0.027

Abbreviations: PPCs, Postoperative Pulmonary Complications; non-PPCs, no Postoperative Pulmonary Complications; CRCs, red cell suspension; SBT, spontaneous breath trial; SD, Standard Deviation; ABG, Arterial Blood Gas Analysis; pH, hydrogen ion concentration; PaCO_2_, arterial carbon dioxide partial pressure; PaO_2_, arterial oxygen partial pressure; WBC, white blood cell; CRP, C-reactive protein; PCT, Procalcitonin

* Red cell suspension and Plasma are not included in the colloidal solution

** All drugs were used intraoperatively. The total dose is the drug dose used throughout the procedure

***The first blood gas analysis during surgery

**** All drugs were used in the ICU after surgery. The total dose is the total amount of drugs used by the patients during the ICU stay

### Increased diaphragm echodensity as a risk factor for PPCs

**Fig. 2:**
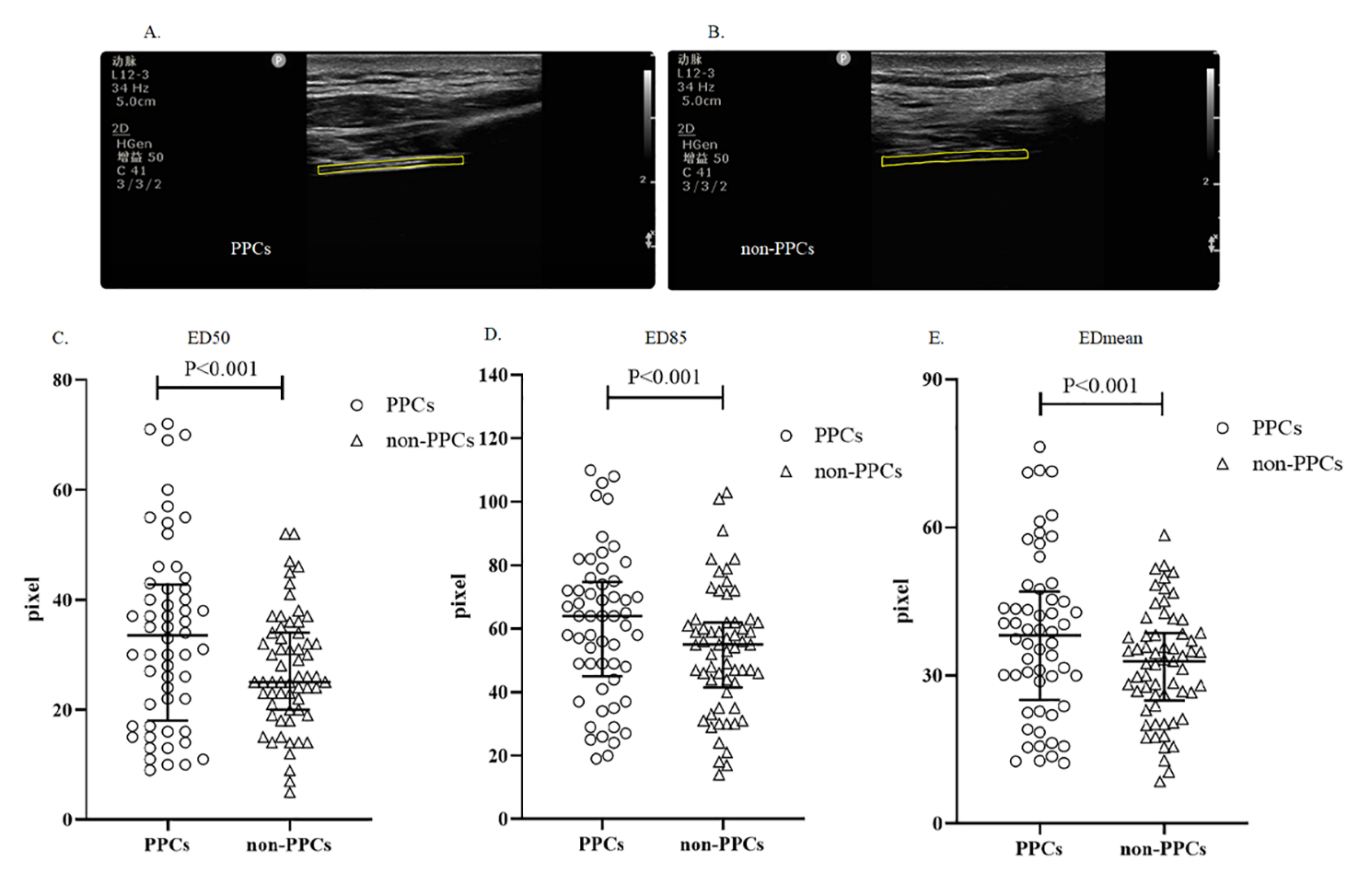
Echodensity histograms of patients at ICU when they underwent SBT (spontaneous breathing trial). (A) The Diaphragm ultrasound image of a patient with PPCs. (B) The Diaphragm ultrasound image of a patient with non-PPCs. (C) Differences in ED50 between PPCs and non-PPCs group; (D) Differences in ED85 between PPCs and non-PPCs group; (E) Differences in EDmean between PPCs and non-PPCs group (the round represents PPCs group; the diamond represents non-PPCs). PPCs: Postoperative Pulmonary Complications; Non-PPCs: none Postoperative Pulmonary Complications

We measured diaphragm echodensity for all participants according to our protocol (Additional Fig. 1). The average difference in ED50 between analyzers was 1.63 (limits of agreement − 17 to 20, *p* = 0.158), which in ED85 was 3.24 (limits of agreement − 32 to 38), and which in EDmean was 1.24 (limits of agreement − 19 to 22) (Additional Fig. 2A, B, C, D). The average difference in ED50 between images within the same analyzer was 0.30 (limits of agreement − 8–8, *p* = 0.700), which in ED85 was − 0.14 (limits of agreement − 13 to 13), and which in EDmean was − 0.07 (limits of agreement − 7 to 7) (Additional Fig. 2E, F, G, H). Within a single respiratory cycle, the ED50 measured by the same analyzer that differed between end-expiration and end-inspiration was 0.50 (limits of agreement − 7 to 8, *p* = 0.476), the ED85 was 2.14 (limits of agreement − 12 to 17) and the EDmean was 1.94 (limits of agreement − 4 to 8) (Additional Fig. 2I, J, K, M). Patients who developed PPCs exhibited a higher ED50 (35.00 [22.00–44.00]) in the PPCs group vs. 26.00 (21.00–35.00) in the non-PPCs group, p < 0.001], a higher ED85 [64.00 (49.00–76.00) in the PPCs group vs. 55.00 (44.00–63.00) in the non-PPCs group, p < 0.001] and a higher EDmean [39.32 (29.88–48.37) in the PPCs group vs. 33.98 (26.14–41.37) in the non-PPCs group, p < 0.001] (Fig. 2).

**Fig. 3:**
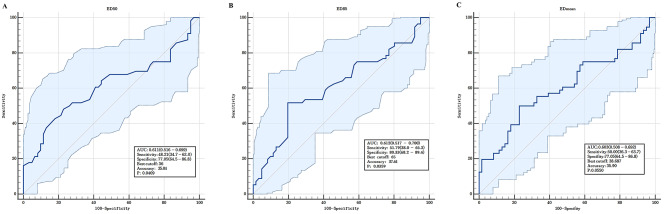
Receiver operator curve (ROC) to assess the ability of Diaphragm echodensity to predict PPCs. (A) represents ED50; (B) represents ED85; (C) represents EDmean.

In ROC curve analysis, the area under the curve of ED50 for predicting PPCs was 0.611 (Fig. 3A). The area under the curve of EDmean for predicting PPCs was 0.603 (Fig. 3B). The area under the curve of ED85 for predicting PPCs was 0.612 (Fig. 3C). The optimal ED50 cutoff value for predicting the occurrence of PPCs was 36. The patients were then categorized according to the optimal ED50 cutoff value: high-risk group (ED50 > 36, n = 35) and low-risk group (ED50 ≤ 36, n = 82). Baseline characteristics data of the two groups were compared in Additional Table 4.

In univariate logistic regression analysis of the occurrence of PPCs, the following factors were associated with PPCs: older (OR, 1.034 [95% CI, 1.030–1.038]), emergency surgery (OR, 5.030 [95% CI, 4.508–5.613]), higher APHACHE II score (OR, 1.060 [95% CI, 1.053–1.067]), higher FiO2 at ICU admission (OR, 1.301 [95% CI, 1.264–1.339]), and higher diaphragm echodensity (e.g., EDmean [OR, 1.032 (95% CI, 1.028–1.036)], ED50(OR, 1.037 [95% CI, 1.033–1.041]), ED85 (OR, 1.018[95% CI, 1.016–1.020]). In multivariable logistic regression analysis of the occurrence of PPCs, higher diaphragm echodensity was also associated with the occurrence of PPCs: model 1: EDmean (OR, 1.026 [95% CI, 1.022–1.029], p *<* 0.001], ED50 (OR, 1.032 [95% CI, 1.027–1.036], *p* < 0.001), ED85 (OR, 1.014 [95% CI, 1.012–1.017], *p* < 0.001); model 2: EDmean (OR, 1.027 [95% CI, 1.022–1.031], *p* < 0.001), ED50 (OR, 1.034 [95% CI, 1.030–1.039], *p* = 0.002), ED85 (OR, 1.013 [95% CI, 1.011–1.016], *p* < 0.001); model 3: EDmean (OR, 1.023 [95% CI, 1.018–1.029], *p* < 0.001), ED50 (OR, 1.031 [95% CI, 1.026–1.037], *p* < 0.001), ED85 (OR, 1.011 [95% CI, 1.008–1.014)], *p* < 0.001). Other independent variables related to PPCs were shown in Table 3.

**Table 3:** Results from univariate and multivariate logistic regression analysis to test the risk of PPCs with diaphragm echodensity

Variable	Unadjusted OR (95%CI)	Unadjusted P value	Model 1*				Model 2*				Model 3*				
			**regression coefficient**	**SE**	**Odds Ratio (95%CI)**	**P value**	**regression coefficient**	**SE**	**Odds Ratio(95%CI)**	**P value**		**regression coefficient**	**SE**	**Odds Ratio(95%CI)**	**P value**
Diaphragm echodensity															
EDmean	1.032 (1.028–1.036)	< 0.001	0.026	0.002	1.026 (1.022–1.029)	< 0.001	0.026	0.002	1.027 (1.022–1.031)	< 0.001		-0.061	-0.119	1.023 (1.018–1.029)	< 0.001
ED50	1.037 (1.033–1.041)	< 0.001	0.031	0.002	1.032 (1.027–1.036)	< 0.001	0.034	0.002	1.034 (1.030–1.039)	0.002		0.031	0.003	1.031 (1.026–1.037)	< 0.001
ED85	1.018 (1.016–1.020)	< 0.001	0.014	0.001	1.014 (1.012–1.017)	< 0.001	0.013	0.001	1.013 (1.011–1.016)	< 0.001		0.011	0.002	1.011 (1.008–1.014)	< 0.001
Age**	1.034 (1.030–1.038)	< 0.001	0.023	0.002	1.023 (1.019–1.027)	< 0.001	0.012	0.002	1.012 (1.008–1.017)	< 0.001		0.043	0.003	1.044 (1.038–1.050)	< 0.001
Emergency surgery**	5.030 (4.508–5.613)	< 0.001	1.584	0.058	4.873 (4.343–5.467)	< 0.001	1.245	0.072	3.473 (3.016–3.998)	< 0.001		1.259	0.090	3.522 (2.952–4.202)	< 0.001
APHACHE II score**	1.060(1.053–1.067)	< 0.001					0.026	0.004	1.027 (1.018–1.035)	< 0.001		0.027	0.005	1.028 (1.018–1.038)	< 0.001
FiO_2_ **	1.301 (1.264–1.339)	< 0.001										0.370	0.023	1.447 (1.384–1.513)	< 0.001
WBC**	1.152 (1.139–1.165)	< 0.001										0.182	0.008	1.199 (1.182–1.217)	< 0.001
PCT**	1.229 (1.190–1.269)	< 0.001										-0.024	0.008	0.976 (0.961–0.991)	0.002
ASA class**															
Grade 2	1 (Ref)						1 (Ref)	1 (Ref)	1 (Ref)			1 (Ref)	1 (Ref)	1 (Ref)	
Grade 3	6.467 (5.794–7.218)	< 0.001					1.237	0.066	3.446 (3.025–3.925)	< 0.001		0.887	0.073	2.428 (2.104–2.802)	< 0.001
Grade 4	1.712 (1.382–2.127)	< 0.001					-0.599	0.139	0.549 (0.419–0.721)	< 0.001		-2.130	0.205	0.119 (0.079–0.178)	< 0.001

Abbreviations: PPCs, Postoperative Pulmonary Complications; non-PPCs, no Postoperative Pulmonary Complications; APACHE II, Acute Physiology and Chronic Health Evaluation; ASA class, American Society of Anesthesiologists class; WBC, white blood cell. CRP, C-reactive protein; PCT, Procalcitonin; ICU, intensive care units; CI, Confidence interval

*Model 1 was adjusted for age, emergency surgery and diaphragm echodensity. Model 2 was adjusted for age, emergency surgery, APHACHE II score, ASA class and diaphragm echodensity. Model 3 was adjusted for age, emergency surgery, APHACHE II score, ASA class, FiO_2_, WBC, PCT and diaphragm echodensity. Only variables with p < 0.05 are reported in models above

** There is collinearity among ED50, ED85 and EDmean and cannot be included in the same model. In Model 1, 2, and 3, the results for these variables were generated by only entering the ED50 into the multivariable-adjusted model. Among them, FiO_2,_ WBC and PCT were obtained at ICU admission

### Diaphragm echodensity and its relationship to ICU outcomes

In the high-risk group (ED50 > 36), 15 (42.86%) patients received COT, four (11.43%) patients received HFNC, and 16 (45.71%) patients received NIPPV. In this group, 24 (68.57%) patients developed PPCs, which was significantly different between the low-risk group and the high-risk group (*p* = 0.003) (Table 4). After multivariate-adjusted analyses, there was still a significant difference between the two groups (*p* = 0.003). Compared with the low-risk group, the high-risk group had higher incidences of PPCs in unadjusted (*p* = 0.003) and multivariate-adjusted analyses (*p* = 0.003). There were no significant differences in VFD-28 (24.61 ± 3.99 in the low-risk group vs. 25.65 ± 3.71 in the high-risk group, *p* = 0.173), length of ICU stay (8.03 ± 7.29 in the low-risk group vs. 5.79 ± 5.63 in the high-risk group, *p* = 0.107), length of hospital stay (20.25 ± 21.33 in the low-risk group vs. 16.09 ± 12.10 in the high-risk group, *p* = 0.279), or mortality rate (*p* = 0.294)

**Table 4:** Diaphragm Echodensity and ICU outcomes

outcome	All (n = 117)	Study group		p value
		**Low-risk (≤ 36, n = 82)**	**High-risk (**≥ **36, n = 35)**	
VFD-28, mean (SD), day	25.35 (3.78)	25.60 (3.77)	24.71 (3.88)	0.253
Mortality, n (%)	6.00 (5.13)	2.00 (2.44)	4.00 (11.43)	0.065
Length of hospital stay, mean (SD), day	17.37 (15.49)	17.09 (14.7)	18.03 (17.61)	0.765
Length of ICU stay, mean (SD), day	6.48 (6.21)	5.98 (5.83)	7.66 (7.05)	0.183
Ventilatory support after extubation, n (%)				0.047
COT	68.00 (58.12)	53.00 (64.63)	15.00 (42.86)	
HFNC	14.00 (11.97)	10.00 (12.2)	4.00 (11.43)	
NIPPV	35.00 (29.91)	19.00 (23.17)	16.00 (45.71)	
Reintubation, n (%)	7.00 (5.98)	4.00 (4.88)	3.00 (8.57)	0.426
PPCs, n (%)	56.00 (47.86)	32.00 (39.02)	24.00 (68.57)	0.003
Multivariate-adjusted of PPCs, n (%) *	56.00 (47.86)	32.00 (39.02)	24.00 (68.57)	0.003
Respiratory failure, n (%)	4.00 (3.42)	4.00 (4.88)	0.00 (0.00)	
Respiratory infection, n (%)	36.00 (30.77)	21.00 (25.61)	15.00 (42.86)	
Pleural effusion, n (%)	27.00 (23.08)	22.00 (26.83)	5.00 (14.29)	
Atelectasis, n (%)	38.00 (32.48)	22.00 (26.83)	18.00 (51.43)	
Bronchospasm, n (%)	2.00 (1.71)	1.00 (1.22)	1.00 (2.86)	

Abbreviations: VFD-28, Ventilation Free Day of 28 days; ICU, intensive care unit; PPCs, Postoperative Pulmonary Complications; non-PPCs, no Postoperative Pulmonary Complications; COT, conventional oxygen therapy; HFNC, high flow nasal cannula; NIPPV, noninvasive positive pressure ventilation

*The multivariate-adjusted of PPCs was adjusted for age, emergency surgery, APHACHE II score, ASA class, FiO2, WBC and PCT.

## Discussion

In this preliminary description of diaphragm echodensity in patients following major abdominal surgery, we found that obtaining diaphragm echodensity measurements was feasible and highly reproducible. Compared with patients without PPCs, patients with PPCs had a higher value of diaphragm echodensity, and the increase in echodensity was related to the occurrence of PPCs. Thus, diaphragm echodensity can be used as an early and simple marker to predict the occurrence of PPCs.

A previous study also found that the technique used to measure diaphragm echodensity was feasible [9]. In our study, this technique proved highly feasible and yielded results with acceptable reproducibility within and between analyzers. Echodensity was not significantly influenced by the timing of image acquisition in the respiratory cycle. Most previous studies have used the mean value of the grayscale intensity as the principal marker of echodensity [6, 14], but the distribution of grayscale values was skewed. To identify a single parameter that best reflects the magnitude of echodensity, we included EDmean, ED50, and ED85. As the distribution median is the simplest and most familiar measurement, we chose to use ED50 as the primary measure of echodensity in our analysis

It has been reported that the reduced diaphragm capacity contributes to pulmonary complications in critically ill patients [13]. Compared with diaphragm thickness, thickness score, and mobility, diaphragm echodensity can essentially reflect muscle capacity, including injury of the diaphragm bundle. Healthy muscle tissue usually appears dark and almost black because it contains little fibrous tissue with minimal sound refection. In critically ill patients with muscle inflammation, necrosis, weakness, and the injury of the muscle bundle, replacement of muscle with fat or fibrous tissue increases echodensity, and muscle appears ‘brighter’ [15]. Therefore, diaphragm echodensity could reflect the injury of the diaphragm caused by major abdominal surgery, and patients with increased echogenicity of the diaphragm may be more likely to develop PPCs. The grayscale value of diaphragm echodensity was used to identify whether the patient was at high risk of developing PPCs after the operation to intervene as quickly as possible

Comparing the predictive effect of several scoring systems, we found the ARISCAT score had a better predictive effect. However, all scores only included preoperative and intraoperative indicators, without taking injury to the diaphragm caused by the surgical procedure into account. As a new predictive index, diaphragm echodensity is simple yet more comprehensive as it is affected by factors such as age, comorbidities, and surgical trauma. Diaphragm echodensity may be used as a comprehensive, novel, and convenient indicator to predict the occurrence of PPCs. Therefore, we propose collecting ultrasound images of the diaphragm during SBT to predict the occurrence of PPCs according to the grayscale value

This study has several important limitations. This study is a proof of concept and that the results obtained from this study cannot be used nor generalized to other case studies obtained with other ultrasound scanners or with different settings (critical pre and post processing-settings). These are first-order analyzes and therefore totally dependent on the machine pre-settings. Standardizing the pre-settings is a crude attempt to reduce the variability of the results, for example adjustable parameters such as gain or depth can affect the grayscale value of the image produced [16]. The angle of the probe, the pressure of the probe on the body, and the subcutaneous fat tissue or edema between the probe and the muscle are all considered potential factors affecting image processing, thus generating the grayscale value of the image [17]. Therefore, the solution to be followed will be to develop second order analyzes which are relatively independent of the ultrasound pre-settings [18]. We only acquired data from the right diaphragm. However, according to expert consensus, in patients without unilateral diaphragmatic pathology, it can be considered that the pathophysiology and function of the left and right diaphragms are the same, and only the right diaphragm can be collected [19]. Abdominal surgical wounds prevented the collection of diaphragm mobility in all patients. Therefore, we cannot verify whether diaphragm mobility is related to the increase in diaphragm echodensity. In addition, we only collected the ultrasound data of patients during SBT, which could not dynamically reflect the changes in the echodensity of the diaphragm after abdominal surgery

## Conclusion

Diaphragm echodensity can be measured feasibly and reproducibly in mechanically ventilated patients, and increased diaphragm echodensity is related to the occurrence of PPCs in patients after major abdominal surgery. Diaphragm echodensity merits further investigation as a potential clinically relevant marker of muscle injury during critical illness

## Electronic supplementary material

Below is the link to the electronic supplementary material.

Supplementary Material 1

Supplementary Material 2

Supplementary Material 3 Supplementary Material 3

Supplementary Material 4 Supplementary Material 4

Supplementary Material 5

Supplementary Material 6

Supplementary Material 7

## Data Availability

The datasets used and/or analyzed during the current study are available from the corresponding author on reasonable request.

## Abbreviations

APACHE II: Acute Physiology and Chronic Health Evaluation
ARISCAT score: Assess Respiratory Risk in Surgical Patients in Catalonia score
ASA class: American Society of Anesthesiologists class
ABG: Arterial Blood Gas Analysis
A/C(VC): Volume Assist/Control Ventilation
A/C(PC): Pressure Assist/Control Ventilation
APTT: Activated partial thromboplastin time
BMI: Body Mass Index
COPD: chronic obstructive pulmonary disease
CI: Confidence interval
COT: conventional oxygen therapy
CRCs: red cell suspension
CRP: C-reactive protein
Ca^2+^: calcium
DBP: diastolic blood pressure
ED50: diaphragm echodensity at 50th percentile of the total pixels
ED85: diaphragm echodensity at 85th percentile of the total pixels
EDmean: mean of the total diaphragm echodensity pixels
Glu: Glucose
HFNC: high flow nasal cannula
HR: Heart Rate
HCT: Red blood cell specific volume
IQR: interquartile range
IL-6: Interleukin-6
ICU: intensive care unit
K^+^: Potassium
Na^+^: sodium
NIPPV: noninvasive positive pressure ventilation
non-PPCs: no Postoperative Pulmonary Complications
PPCs: Postoperative Pulmonary Complications
pH: hydrogen ion concentration
PaCO_2_: arterial carbon dioxide partial pressure
PaO_2_: arterial oxygen partial pressure
PCT: Procalcitonin
PSV: Pressure Support Ventilation
PEEP: Positive end expiration pressure
PT: prothrombin time
RR: Respiratory rate
SBT: spontaneous breath trial
SD: Standard Deviation
SBP: systolic blood pressure
SIMV: Synchronized Intermittent Mandatory Ventilation
VFD-28: Ventilation Free Day of 28 days
WBC: white blood cell
