# Supplementary material for: Increased diaphragm echodensity correlates with postoperative pulmonary complications in patients after major abdominal surgery: a prospective observational study
Source: BMC Pulm Med. 2022 Nov 4;22:400. doi: 10.1186/s12890-022-02194-6 (PMC9636692; doi:10.1186/s12890-022-02194-6)
Supplement: Supplementary file 1 — Supplementary Material 1 [file 12890_2022_2194_MOESM1_ESM.docx]

| **eTable 2.** **Additional Intraoperative Data** | | | | |
| --- | --- | --- | --- | --- |
| **outcome** | **All (n=117)** | **Study group** | | **P value** |
|  |  | **PPCs (n=56)** | **Non-PPCs (n=61)** |  |
| Blood loss, mean (SD), ml | 414.15 (815.46) | 348.57 (534.5) | 479.51 (1017.79) | 0.392 |
| Use of other drugs, mean (SD) | | | | |
| Desflurane, ml | 56.15 (50.91) | 49.07 (30.63) | 61.46 (64.76) | 0.603 |
| Sevoflurane, ml | 64.54 (38.13) | 64.14 (25.11) | 64.8 (47.15) | 0.927 |
| Propofol, ml | 193.86 (288.67) | 176.98 (273.82) | 210.84 (307.05) | 0.551 |
| Midazolam, ml | 2.03 (0.83) | 2.11 (1.01) | 1.95 (0.70) | 0.351 |
| Dexamethasone, ml | 7.75 (2.14) | 8.75 (2.09) | 7.25 (2.16) | 0.180 |
| ABG at operating room admission, mean (SD) | | | | |
| pH | 7.4 (0.1) | 7.38 (0.13) | 7.41 (0.04) | 0.113 |
| PaCO_2_, mmHg | 37.42 (6.13) | 37.4 (6.78) | 37.45 (5.56) | 0.962 |
| PaO_2_, mmHg | 220.76 (90.69) | 208.75 (91.3) | 232.31 (90.34) | 0.193 |
| HCT | 33.74 (8.56) | 32.73 (8.77) | 34.73 (8.42) | 0.227 |
| Na^+^, mmol/L | 135.86 (4.12) | 133.25 (19.02) | 135.97 (3.7) | 0.301 |
| K^+^, mmol/L | 3.5 (0.55) | 3.51 (0.85) | 3.41 (0.34) | 0.428 |
| Ca^2+^, mmol/L | 1.26 (0.55) | 1.27 (0.75) | 1.18 (0.37) | 0.416 |
| Glu, mmol/L | 6.95 (3.22) | 7.76 (3.51) | 6.02 (2.83) | 0.005 |
| Lactate, mmol/L | 1.56 (1.96) | 1.62 (2.7) | 1.29 (0.52) | 0.388 |
| Hemoglobin, g/L | 106.73 (28.93) | 98.97 (33.58) | 112.4 (26.17) | 0.022 |
| HCO_3_, mmol/L | 22.8 (3.73) | 21.77 (5.65) | 23.25 (2.39) | 0.080 |
| Base excess | -7.42 (57.67) | -13.83 (82.17) | -1.12 (2.08) | 0.261 |

Abbreviations: PaCO_2_, arterial carbon dioxide partial pressure; PaO_2_, arterial oxygen partial pressure; HCT, Red blood cell specific volume; Na^+^, sodium; K^+^, Potassium; Ca^2+^, calcium; Glu: Glucose.
