# Supplementary material for: Increased diaphragm echodensity correlates with postoperative pulmonary complications in patients after major abdominal surgery: a prospective observational study
Source: BMC Pulm Med. 2022 Nov 4;22:400. doi: 10.1186/s12890-022-02194-6 (PMC9636692; doi:10.1186/s12890-022-02194-6)
Supplement: Supplementary file 2 — Supplementary Material 2 [file 12890_2022_2194_MOESM2_ESM.docx]

| **eTable 3.** **Additional Postoperative Data** | | | | |
| --- | --- | --- | --- | --- |
| **outcome** | **All (n=117)** | **Study group** | | **p value** |
|  |  | **PPCs (n=56)** | **Non-PPCs (n=61)** |  |
| Mechanical ventilation, mean (SD)* | | | | |
| Ventilatory mode, n (%) | | | | 0.436 |
| A/C(VC) | 99.00 (84.62) | 49.00 (87.5) | 50.00 (81.97) |  |
| A/C(PC) | 7.00 (5.98) | 4.00 (7.14) | 3.00 (4.92) |  |
| SIMV | 2.00 (1.71) | 1.00 (1.79) | 1.00 (1.64) |  |
| PSV | 9.00 (7.69) | 2.00 (3.57) | 7.00 (11.48) |  |
| RR, bpm | 12.31 (1.13) | 12.22 (1.07) | 12.41 (1.22) | 0.378 |
| Tidal volume, ml | 432.62 (40.22) | 434.71 (39.06) | 430.20 (42.31) | 0.577 |
| PEEP, cmH2O | 6.29 (2.06) | 6.65 (2.40) | 5.98 (1.68) | 0.088 |
| FiO_2_ at ICU admission | 34.28 (4.96) | 42.68 (6.94) | 38.77 (4.53) | 0.001 |
| FiO_2_ after extubation | 32.82 (14.31) | 35.54 (5.99) | 33.15 (3.55) | 0.011 |
| Vital sign at ICU admission |  |  |  |  |
| Heart rate | 86.04 (19.03) | 87.25 (20.49) | 84.53 (17.77) | 0.446 |
| SBP | 142.75 (9.6) | 137.61 (25.07) | 139.28 (19.11) | 0.685 |
| DBP | 91.75 (5.07) | 73.79 (12.04) | 78.93 (12.94) | 0.029 |
| ABG at ICU admission, mean (SD)* | | | | |
| pH | 7.35 (0.04) | 7.38 (0.05) | 7.36 (0.05) | 0.039 |
| PCO_2_, mmHg | 41.12 (4.95) | 37.07 (6.31) | 39.58 (5.28) | 0.021 |
| PO_2_, mmHg | 125.37 (29.74) | 115.71 (39.97) | 147.11 (35.72) | <0.001 |
| Na^+^, mmol/L | 136.50 (2.00) | 135.43 (4.43) | 135.44 (3.38) | 0.992 |
| K^+^, mmol/L | 3.68 (0.34) | 3.68 (0.45) | 3.73 (0.50) | 0.575 |
| Blood biochemical examination at ICU admission, mean (SD) | | | | |
| Blood platelet, *10^9/L | 170.83 (50.57) | 231.05 (155.81) | 205.98 (104.9) | 0.306 |
| Total bilirubin, μmol/L | 13.37 (6.00) | 28.85 (36.32) | 24.54 (35.06) | 0.514 |
| Glu, mmol/L | 8.49 (2.17) | 8.06 (2.35) | 7.80 (2.95) | 0.606 |
| IL-6, pg/ml | 159.20 (161.15) | 616.50 (1039.97) | 396.39 (790.86) | 0.211 |
| D-dimer, ng/ml | 1.72 (0.83) | 6.53 (6.21) | 4.63 (6.43) | 0.114 |
| APTT, s | 30.07 (2.63) | 31.74 (10.6) | 29.28 (7.25) | 0.153 |
| PT, s | 27.80 (18.70) | 13.37 (2.16) | 13.28 (5.99) | 0.910 |

Abbreviations: A/C(VC), Volume Assist/Control Ventilation; A/C(PC), Pressure Assist/Control Ventilation; SIMV, Synchronized Intermittent Mandatory Ventilation; PSV, Pressure Support Ventilation; RR, Respiratory rate; PEEP, Positive end expiration pressure; HR, Heart Rate; SBP, systolic blood pressure; DBP, diastolic blood pressure; ABG, Arterial Blood Gas Analysis; pH, hydrogen ion concentration; PaCO_2_, arterial carbon dioxide partial pressure; PaO_2_, arterial oxygen partial pressure; Glu, glucose; IL-6, Interleukin-6; APTT, Activated partial thromboplastin time; PT, prothrombin time; Na^+^, sodium; K^+^, Potassium.

* Mechanical ventilation at admission to ICU

**First blood gas analysis after admission to ICU
