# Supplementary figures and images for: Increased diaphragm echodensity correlates with postoperative pulmonary complications in patients after major abdominal surgery: a prospective observational study
Source: BMC Pulm Med. 2022 Nov 4;22:400. doi: 10.1186/s12890-022-02194-6 (PMC9636692; doi:10.1186/s12890-022-02194-6)

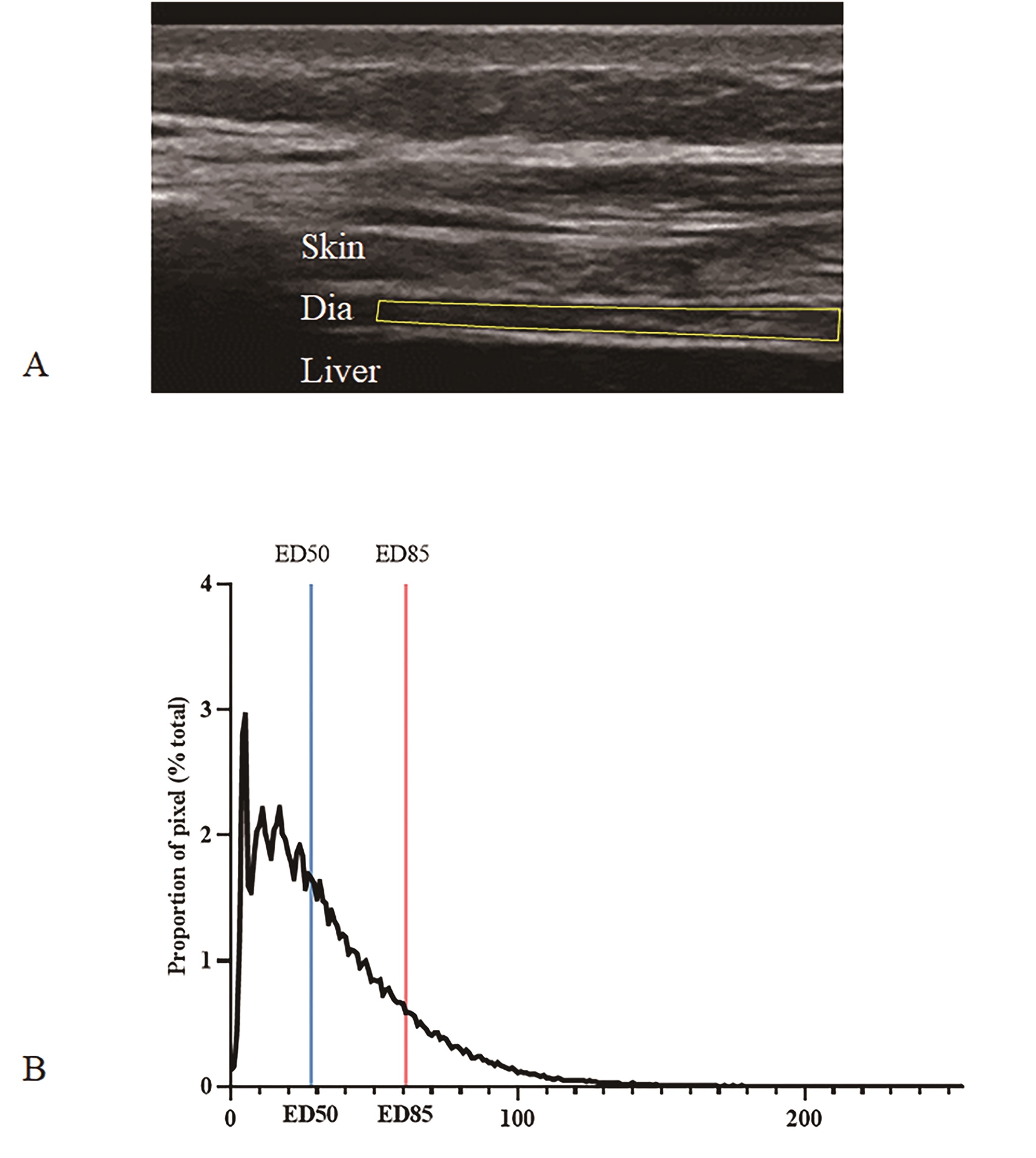

Supplement: Supplementary file 3 — Supplementary Material 3 [file 12890_2022_2194_MOESM3_ESM.jpg]

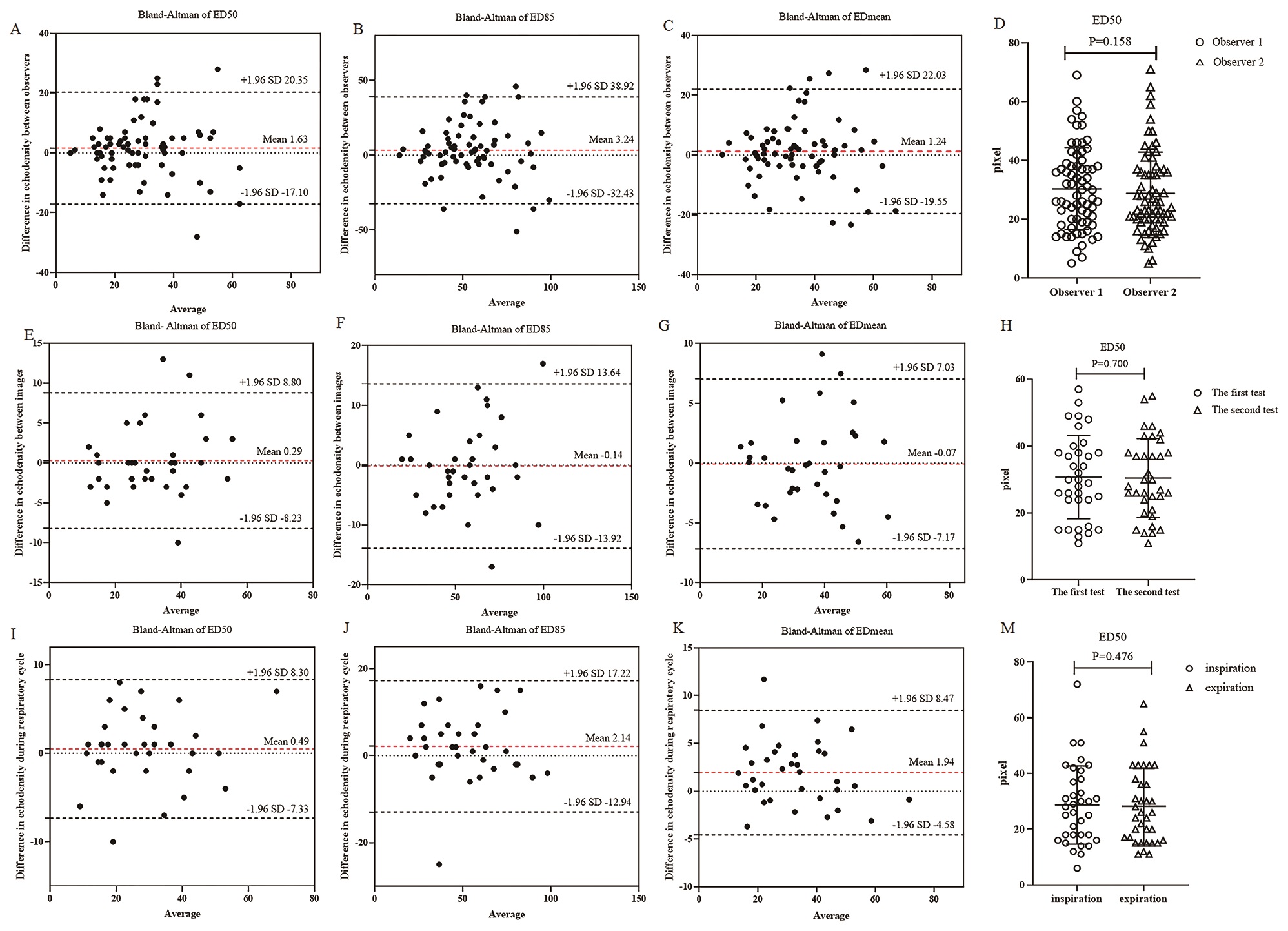

Supplement: Supplementary file 4 — Supplementary Material 4 [file 12890_2022_2194_MOESM4_ESM.jpg]
