# Supplementary material for: Increased diaphragm echodensity correlates with postoperative pulmonary complications in patients after major abdominal surgery: a prospective observational study
Source: BMC Pulm Med. 2022 Nov 4;22:400. doi: 10.1186/s12890-022-02194-6 (PMC9636692; doi:10.1186/s12890-022-02194-6)
Supplement: Supplementary file 5 — Supplementary Material 5 [file 12890_2022_2194_MOESM5_ESM.docx]

| **eTable 1. Postoperative Pulmonary Complications** | |
| --- | --- |
|  | Definition |
| Pulmonary infection | Defined as need of antibiotics for a suspected respiratory infection and one or more of the following criteria: new or changed sputum, new or changed lung opacities, fever and/or white blood cell count>12×10^9^/L |
| Bronchospasm | Defined as Newly detected expiratory wheezing treated with bronchodilators |
| Atelectasis | Defined as lung opacification with a shift of the mediastinum, hilum or hemidiaphragm toward the affected area, and compensatory over-inflation in the adjacent non-atelectatic lung |
| Respiratory failure | Defined as a postoperative PaO_2_< 60 mmHg on room air, a PaO_2_/FiO_2_ ratio < 40 kPa (300 mmHg) or arterial oxyhemoglobin saturation measured with pulse oximetry< 90% and requiring oxygen therapy |
| Pleural effusion | Defined as chest radiograph demonstrating blunting of the costophrenic angle, loss of sharp silhouette of the ipsilateral hemidiaphragm in upright position, evidence of displacement of adjacent anatomical structures or (in supine position) a hazy opacity in one hemithorax with preserved vascular shadows |
| Pneumothorax | Defined as air in the pleural space with no vascular bed surrounding the visceral pleura |
| Aspiration pneumonitis | Defined as acute lung injury after the inhalation of regurgitated gastric contents |
