# Supplementary material for: Increased diaphragm echodensity correlates with postoperative pulmonary complications in patients after major abdominal surgery: a prospective observational study
Source: BMC Pulm Med. 2022 Nov 4;22:400. doi: 10.1186/s12890-022-02194-6 (PMC9636692; doi:10.1186/s12890-022-02194-6)
Supplement: Supplementary file 6 — Supplementary Material 6 [file 12890_2022_2194_MOESM6_ESM.docx]

| **eTable 4: Baseline characteristics of participants based on diaphragm echodensity** | | | | |
| --- | --- | --- | --- | --- |
| **Characteristics** | **All (n=117)** | **Study group** | | |
|  |  | **Low-risk (≤36, n=82)** | **High-risk (>36, n=35)** | **P value** |
| Age, mean (SD), y | 57.57 (15.46) | 55.51 (15.50) | 61.80 (14.71) | 0.044 |
| Male, n (%) | 78.00 (66.67) | 59.00 (70.37) | 19.00 (58.33) | 0.063 |
| BMI, mean (SD) | 22.33 (4.69) | 22.49 (4.04) | 22.44 (4.81) | 0.956 |
| Vital sign at ICU admission |  |  |  |  |
| Heart rate | 86.04 (19.03) | 85.88 (18.65) | 85.77 (20.37) | 0.978 |
| SBP | 142.75 (9.60) | 139.98 (22.23) | 135.00 (21.73) | 0.268 |
| DBP | 91.75 (5.07) | 76.74 (12.00) | 75.77 (14.43) | 0.708 |
| ARISCAT score, mean (SD) | 38.39 (16.74) | 37.11 (17.01) | 42.14 (15.54) | 0.136 |
| ASA class, n (%) | | | | 0.893 |
| Grade 2 | 55.00 (47.01) | 38.00 (46.34) | 17.00 (48.57) |  |
| Grade 3 | 56.00 (47.86) | 39.00 (47.56) | 17.00 (48.57) |  |
| Grade 4 | 6.00 (5.13) | 5.00 (6.10) | 1.00 (2.86) |  |
| APACHE II score, mean (SD) | 13.29 (7.39) | 13.39 (7.74) | 13.06 (6.71) | 0.825 |
| Comorbidity, n (%) | | | |  |
| Cardiovascular disease | 20.00 (17.09) | 13.00 (15.85) | 7.00 (20.00) | 0.585 |
| Diabetes mellitus | 9.00 (7.69) | 7.00 (8.54) | 2.00 (5.71) | 0.884 |
| COPD | 4.00 (3.42) | 2.00 (2.44) | 2.00 (5.71) | 0.736 |
| Hyperlipoidemia | 5.00 (4.27) | 3.00 (3.66) | 2.00 (5.71) | 0.997 |
| Hypertension | 17.00 (14.53) | 11.00 (13.41) | 6.00 (17.14) | 0.600 |
| Active cancer | 49.00 (41.88) | 35.00 (42.68) | 14.00 (40.00) | 0.788 |
| Chronic renal disease | 38.00 (32.48) | 26.00 (31.71) | 12.00 (34.29) | 0.785 |
| Chronic liver disease | 48.00 (41.03) | 35.00 (42.68) | 13.00 (37.14) | 0.577 |
| Chronic bronchitis | 4.00 (3.42) | 1.00 (1.22) | 3.00 (8.57) | 0.148 |
| Type of surgery, n (%) |  |  |  |  |
| Emergency surgery | 42.00 (35.90) | 27.00 (32.93) | 15.00 (42.86) | 0.305 |
| Planned surgical procedure, n (%) |  |  |  | 0.072 |
| Resection of colon, rectum, or small bowel | 19.00 (16.24) | 13.00 (15.85) | 6.00 (17.14) |  |
| Resection of liver, pancreas, or gall bladder | 69.00 (58.98) | 50.00 (60.98) | 19.00 (54.29) |  |
| Resection of stomach | 5.00 (4.27) | 2.00 (2.44) | 3.00 (8.57) |  |
| Other intraperitoneal surgery | 24.00 (20.51) | 18.00 (21.95) | 6.00 (17.14) |  |
| Incision type, n (%) |  |  |  | 0.514 |
| Midline laparotomy | 61.00 (52.14) | 42.00 (51.22) | 19.00 (54.29) |  |
| Bilateral or unilateral subcostal | 23.00 (19.66) | 19.00 (23.17) | 4.00 (11.43) |  |
| Transverse abdominal | 1.00 (0.85) | 1.00 (1.22) | 0.00 (0.00) |  |
| Laparoscopic or lower abdominal | 13.00 (11.10) | 9.00 (10.98) | 4.00 (11.43) |  |
| Other | 19.00 (16.24) | 11.00 (13.41) | 8.00 (22.86) |  |
| Length of procedure, mean (SD), mins | 199.84 (189.01) | 207.27 (187.22) | 181.77 (199.97) | 0.639 |
| Diaphragm activity, mean (SD), cm | 1.10 (0.44) | 1.12 (0.49) | 1.05 (0.28) | 0.395 |
| Diaphragm thickness, mean (SD), cm | 0.23 (0.07) | 0.23 (0.06) | 0.23 (0.08) | 0.806 |
| Intraoperative management | | | | |
| Type of intraoperative fluid, mean (SD), ml | | | | |
| Crystalloid | 1905.25 (1314.06) | 1936.22 (1375.51) | 1815.71 (1210.11) | 0.944 |
| Colloid* | 945.14 (607.14) | 933.02 (636.01) | 978.95 (551.59) | 0.786 |
| blood transfusion | | | | |
| CRCs, mean (SD), u | 6.00 (4.15) | 6.27 (4.28) | 5.15 (4.03) | 0.610 |
| Plasma, mean (SD), ml | 823.44 (806.03) | 931.25 (909.71) | 500.00 (302.37) | 0.720 |
| Use of muscle relaxant, mean (SD), mg | | | | |
| Vecuronium bromide | 6.06 (4.95) | 5.83 (6.36) | 7.00 (0.00) | 0.879 |
| Rocuronium Bromide | 82.96 (33.84) | 70.50 (28.33) | 88.75 (37.04) | 0.196 |
| Atracurium | 27.71 (20.44) | 29.35 (21.72) | 23.36 (16.54) | 0.182 |
| Succinylcholine | 76.79 (18.28) | 82.50 (16.54) | 62.50 (18.93) | 0.072 |
| Use of vasoactive drugs, mean (SD), mg |  |  |  |  |
| Metaraminol | 0.63 (0.63) | 0.73 (0.75) | 0.48 (0.37) | 0.084 |
| Ephedrine | 8.06 (6.68) | 7.94 (6.58) | 8.33 (7.49) | 0.871 |
| Norepinephrine | 1.76 (2.11) | 1.80 (1.99) | 1.66 (2.53) | 0.863 |
| Epinephrine | 101.05 (95.36) | 133.73 (108.53) | 3.00 (0.00) | 0.406 |
| Use of opioids, mean (SD), µg |  |  |  |  |
| Remifentanil | 1463.84 (809.02) | 1457.79 (873.98) | 1451.10 (652.26) | 0.968 |
| Sufentanil | 43.91 (22.76) | 46.65 (24.38) | 39.06 (18.93) | 0.758 |
| Mechanical ventilation, mean (SD) | | | | |
| Ventilatory mode, n (%) | | |  | 0.436 |
| A/C(VC) | 99.00 (84.62) | 49.00 (59.76) | 50.00 (142.86) |  |
| A/C(PC) | 7.00 (5.98) | 4.00 (4.88) | 3.00 (8.57) |  |
| SIMV | 2.00 (1.71) | 1.00 (1.22) | 1.00 (2.86) |  |
| PSV | 9.00 (7.69) | 2.00 (2.44) | 7.00 (20.00) |  |
| RR, bpm | 12.31 (1.13) | 12.31 (1.23) | 12.32 (0.94) | 0.960 |
| Tidal volume, ml | 432.62 (40.22) | 435.29 (42.66) | 426.25 (35.45) | 0.299 |
| PEEP, cmH2O | 6.29 (2.06) | 6.26 (2.05) | 6.41 (2.15) | 0.721 |
| FiO_2_ at ICU admission | 34.28 (4.96) | 40.24 (3.76) | 41.57 (9.61) | 0.433 |
| FiO_2_ after extubation | 32.82 (14.31) | 33.91 (4.38) | 35.17 (6.20) | 0.214 |
| ABG at ICU admission, mean (SD) |  |  |  |  |
| pH | 7.35 (0.04) | 7.37 (0.05) | 7.36 (0.05) | 0.279 |
| PCO_2_, mmHg | 41.12 (4.95) | 38.57 (5.56) | 37.93 (6.72) | 0.593 |
| PO_2_, mmHg | 125.37 (29.74) | 133.51 (41.04) | 128.73 (40.67) | 0.564 |
| Lactate, mmol/L | 1.68 (0.42) | 2.42 (2.04) | 2.36 (1.91) | 0.874 |
| Na^+^, mmol/L | 136.50 (2.00) | 135.85 (4.03) | 134.46 (3.46) | 0.079 |
| K^+^, mmol/L | 3.68 (0.34) | 3.71 (0.51) | 3.69 (0.39) | 0.803 |
| Blood biochemical examination at ICU admission, mean (SD) | | | | |
| WBC, 10^9/L | 10.96 (4.66) | 10.56 (5.30) | 11.23 (5.58) | 0.543 |
| Hemoglobin, mg/L | 111.50 (4.57) | 114.49 (26.6) | 107.31 (25.99) | 0.181 |
| Blood platelet, *10^9/L | 170.83 (50.57) | 219.46 (108.88) | 214.51 (175.97) | 0.853 |
| Total bilirubin, μmol/L | 13.37 (6.00) | 25.75 (36.10) | 28.60 (34.75) | 0.693 |
| Serum albumin, g/L | 33.28 (2.64) | 32.39 (7.19) | 35.21 (7.22) | 0.037 |
| Serum creatinine, μmol/L | 217.17 (325.87) | 77.15 (49.65) | 111.51 (171.26) | 0.251 |
| CRP, mg/L | 61.01 (99.48) | 58.67 (94.05) | 74.58 (93.75) | 0.408 |
| PCT, ng/ml | 1.39 (3.82) | 0.44 (0.79) | 3.36 (8.74) | 0.056 |
| IL-6, pg/ml | 159.20 (161.15) | 459.32 (927.1) | 599.77 (912.11) | 0.460 |
| D-dimer, ng/ml | 1.72 (0.83) | 5.22 (6.24) | 6.37 (6.69) | 0.388 |
| APTT, s | 30.07 (2.63) | 29.67 (6.35) | 32.45 (13.54) | 0.266 |
| PT, s | 27.8 (18.7) | 13.36 (5.18) | 13.23 (2.31) | 0.890 |
| Glu, mmol/L | 8.49 (2.17) | 7.98 (2.85) | 7.80 (2.23) | 0.750 |
| ABG when the SBT ends, mean (SD)* |  |  |  |  |
| pH, mmHg | 7.38 (0.05) | 7.41 (0.05) | 7.36 (0.15) | 0.075 |
| PaO_2_, mmHg | 112.60 (13.20) | 222.73 (93.75) | 215.81 (85.56) | 0.731 |
| PaCO_2_, mmHg | 41.95 (2.95) | 37.10 (5.57) | 38.21 (7.44) | 0.396 |

Abbreviations: BMI, Body Mass Index; ARISCAT score, Assess Respiratory Risk in Surgical Patients in Catalonia score; APACHE II, Acute Physiology and Chronic Health Evaluation; ASA class, American Society of Anesthesiologists class; COPD, chronic obstructive pulmonary disease; HR, Heart Rate; SBP: systolic blood pressure; DBP, diastolic blood pressure; CRCs, red cell suspension; SBT, spontaneous breath trial; SD, Standard Deviation; ABG, Arterial Blood Gas Analysis; WBC, white blood cell. CRP, C-reactive protein; PCT, Procalcitonin.;

* Red cell suspension and Plasma are not included in the colloidal solution
