# Supplementary material for: Increased diaphragm echodensity correlates with postoperative pulmonary complications in patients after major abdominal surgery: a prospective observational study
Source: BMC Pulm Med. 2022 Nov 4;22:400. doi: 10.1186/s12890-022-02194-6 (PMC9636692; doi:10.1186/s12890-022-02194-6)
Supplement: Supplementary file 7 — Supplementary Material 7 [file 12890_2022_2194_MOESM7_ESM.docx]

Additional figure legends

Additional Figure 1. Method of analysis of the diaphragm echodensity. (A) Example of a diaphragm ultrasound image (Dia: diaphragm). The yellow rectangle delineates the diaphragm area (excluding the pleural and peritoneal membranes). (B) Example of a histogram in a patient representing the proportion of pixels (percentage of the total pixels) at each grayscale intensity of the diaphragm. The two right straight lines represent the grayscale intensity at 50th (ED50, blue line) and 85th (ED85, red line) percentile of the total pixels.

Additional Figure 2. Paired-samples t test and Bland-Altman plot of repeated measurements of diaphragm echodensity. The analyses were performed with ED50, ED85 and EDmean (i.e grayscale intensity at 50th percentile of the total pixels = ED50). The red dashed line indicates bias, the black dashed lines indicate both limits of agreement. The x-axis shows the mean of two values. The y-axis shows the difference between ED50(including ED85 and EDmean) of these values. Between-analyzer reproducibility of echogenicity (measurement on one image, two analyzers, n=70 images): (A) ED50: bias = 1.63, limits (-17.10; 20.35); (B) ED85: bias = 3.24, limits (-32.43; 38.92); (C) EDmean: bias = 1.24, limits (-19.55; 22.03); (D) Paired-samples t test of echodensity: p=0.158. Between-image reproducibility of echogenicity (measurement on two separate images collected on the same patient on the same day, single analyzer, n=35 images): (E) ED50: bias = 0.29, limits (-8.23; 8.80); (F) ED85: bias = -0.14, limits (-13.92; 13.64); (G) EDmean: bias = -0.07, limits (-7.17; 7.03); (H) Paired-samples t test of echodensity: p=0.700. Reproducibility of echodensity at end-expiration and end-inspiration (2 measurements on the same respiratory cycle, single analyzer，n=35 images.): (I) ED50: bias = 0.49, limits (-7.33; 8.30); (J) ED85: bias = 2.14, limits (-12.94; 17.22); (K) EDmean: bias = 1.94, limits (-4.58; 8.47); (M) Paired-samples t test of echodensity: p=0.476.
